# Computational and experimental elucidation of *Plasmodium falciparum* phosphoethanolamine methyltransferase inhibitors: Pivotal drug target

**DOI:** 10.1371/journal.pone.0221032

**Published:** 2019-08-22

**Authors:** Jagbir Singh, Sonam Vijay, Rani Mansuri, Ritu Rawal, Kavita Kadian, Ganesh Chandra Sahoo, Mahesh Kumar, Arun Sharma

**Affiliations:** 1 Protein Biochemistry and Structural Biology, ICMR-National Institute of Malaria Research, New Delhi, India; 2 Department of Pharmaceutical Sciences, Maharshi Dayanand University, Rohtak, India; 3 School of Pharmaceutical Sciences, ApeejayStya University, Gurugram, India; 4 Department of Biomedical Sciences, Rajendra Memorial Research Institute, Patna, India; University of California San Francisco, UNITED STATES

## Abstract

**Introduction:**

*Plasmodium falciparum* synthesizes phosphatidylcholine for the membrane development through serine decarboxylase–phosphoethanolamine methyltransferase pathway for growth in human host. Phosphoethanolamine-methyltransferase (*Pf*PMT) is a crucial enzyme for the synthesis of phosphocholine which is a precursor for phosphatidylcholine synthesis and is considered as a pivotal drug target as it is absent in the host. The inhibition of *Pf*PMT may kill malaria parasite and hence is being considered as potential target for rational antimalarial drug designing.

**Methods:**

In this study, we have used computer aided drug designing (CADD) approaches to establish potential *Pf*PMT inhibitors from Asinex compound library virtually screened for ADMET and the docking affinity. The selected compounds were tested for *in-vitro* schizonticidal, gametocidal and cytotoxicity activity. Nontoxic compounds were further studied for *Pf*PMT enzyme specificity and antimalarial efficacy for *P*. *berghei* in albino mice model.

**Results:**

Our results have identified two nontoxic *Pf*PMT competitive inhibitors ASN.1 and ASN.3 with better schizonticidal and gametocidal activity which were found to inhibit *Pf*PMT at IC_50_ 1.49μM and 2.31μM respectively. The promising reduction in parasitaemia was found both in orally (50 & 10 mg/kg) and intravenous (IV) (5& 1 mg/kg) however, the better growth inhibition was found in intravenous groups.

**Conclusion:**

We report that the compounds containing Pyridinyl-Pyrimidine and Phenyl-Furan scaffolds as the potential inhibitors of *Pf*PMT and thus may act as promising antimalarial inhibitor candidates which can be further optimized and used as leads for template based antimalarial drug development.

## Introduction

Despite several advances and progresses for the control of malaria, it is still an important global health problem. There is no significant fall in malaria burden globally from an estimated 239 cases in 2010 to 219 million new malaria cases in 2017 and thousands of death by malaria occurred globally[1]. It is surprising that the 80% of the burden is solely found in India and sub Saharan African countries as per WHO report 2018 [1]. In a current scenario, malaria treatment is relied on very few drugs. Spread of resistance to first line antimalarial drugs including artemisinin based combination therapy calls for the search and novel drug development [2]. Efforts are required to focus on novel inhibitor compounds that can specifically target and block parasite growth and transmission of plasmodium parasite.

In plasmodium, lipids play significant roles in various metabolic processes starting from membrane biogenesis to signaling as well being essential for their survival and viability. Phosphatidylcholine (PC) is considered to be the major and essential phospholipid constituent of membranes that play a key role in parasite growth, proliferation and survivals.Biosynthesis of PC has also been found to be essential for survival of many other organisms such as *Leishmania major*, bacteria’s, and eukaryotes [3–5].

Therefore, the pathways of phospholipids biosynthesis are considered to be attractive targets for antimalarial drug discovery. PC synthesis occurs mainly by three routes *de novo* (CDP-choline), CDP- ethanolamine (kennedy pathway) and CDP-diacylglycrol pathway [6–7]. In case of malaria, parasite requires an active production of PC by alternative pathway i.e. serine decarboxylation pathway (SDPM) for synthesis of new membranes required for rapid proliferation of parasite during both intraerythrocytic and gametocyte development [8–9]. In plasmodium, an atypical step is the synthesis of phosphocholine from ethanolamine catalyzed by Phosphoethanolamine methyl transferase (PMT) and absence of this enzyme in mammals making an attractive antimalarial drug target. PMT gene knockout studies have shown complete inhibition of the PC biosynthesis from serine and caused serious defects in parasite survival and multiplication. However, when *Pf*PMT was reintroduced into knock out strain, gametocyte differentiation was restored to wild type level [10]. It is known that *Pf*PMT and plant methyltransferase *(*PEAMT) are homologues but only for three methylation steps i.e. plants PEAMT have two catalytic domains and *Pf*PMT has one catalytic domain. This implies that same catalytic domain is being used for all triple methylations [11–13].Therefore; the inhibition of catalytic domain of *Pf*PMT can lead to complete inhibition of SDPM pathway. Since *Pf*PMT has been found to be expressed throughout the asexual and sexual phases of parasite life cycle and absence of its human orthologues therefore it may be used as target template for development of new antimalarial [14–16].Biochemical studies have demonstrated that human malaria parasite orthologs of PMT have similar structural as well as biochemical properties and inhibition profiles as plasmodium [17].

In the present study, we report the characterization and phylogenetic comparison of *Pf*PMTgene and comparative analysis showed significant identity among SAM-dependent methyltransferase domains and motifs. To delineate this protein as a drug target, we undertook docking studies to identify the compounds inhibitors of *Pf*PMT that were either interacting with crucial amino acids or occupying the catalytic dyad important for triple methylationthrough *in vitro* and *in vivo* efficacy studies in albino mice as well. We have aimed to project these antimalarial inhibitors against PMT which may prevent the function of target protein and synthesis of the phosphatidylcholine not only in *Plasmodium falciparum* but also in other studied *Plasmodium* orthologues. Our present study have identified that the Asinex compounds containing Pyridinyl-yrimidine and compounds containing Phenyl-Furan scaffolds as specific PMT inhibitors which may provide insights into the mechanism of blocking transmission of the malaria infection and may set as an imminent prospect to overcome the problem of antimalarial drug resistance.

## Materials and methods

### Ethical approval

Present study was approved by the Human Ethical Committee of National Institute of Malaria Research (NIMR), New Delhi. Written informed consent was documented and mandatorily sought from each patient prior to the sample collection. Parasite bank, NIMR is well established and routinely maintained. Animal experiments were conducted at Department of Animal Experiments, National JALMA Institute for Leprosy and Other Mycobacterial diseases (ICMR), Agra for ethical approval from the Animal Research Ethics Committee JALMA, Agra, India (Approval Id: JALMA/2018/10).

### Sample collection

*P*. *falciprum* malaria +ve blood samples were collected using filter paper method. Briefly, by finger prick, blood (2–3 drops) of the patients visiting to the malaria Clinic at NIMR, Delhi was collected on 3 mm filter paper strips (Whatman International Ltd. Maidstone, UK). The *Pf* positive was confirmed by screening with microscope and Rapid Diagnostic Test kit Falci Vax (Zephyr Biomedicals, India).

### *Pf*PMT gene amplification

*P*. *falciparum* genomic DNA was isolated from blood samples using a QIAamp DNA Blood Mini Kit.Two set of primers Forward primer 5’ CTTAGAATCCTGTTTTGTTCC 3’ and Reverse primer 5’ CCACTAACTCTTTCATTAGCC 3’), second set (Forward primers 5’ GATCTGGTTTAGGAGGTGGT 3’ and Reverse primers 5’ GGAGGTATGTGTGTGTATTT 3’) were designed using Primer3 based on the nucleotide *Pf*PMT 3D7 isolate and synthesized. cDNA was synthesized from whole RNA isolated from *P*. *falciparum* culture as per manufacturer instructions (Qiagen,Hilden, Germany). *Pf*PMT gene PCR product was gel purified by exosap IT (Fermentas, CA, USA) and cloned in *E*.*coli* DH5α competent cells [18]. Sequencing at First BASE Laboratories SdnBhd **(**Selangor, Malaysia**)** confirmed the positive recombinant clones. DNA sequence chromatogram was further analyzed using FINCHTV and BioEdit tool [19].The amplified nucleotide sequence of Indian isolate of *Pf*PMT gene (Ind_*Pf*PMT) was submitted to NCBI Gen Bank and available with the accession numbers **KX755406** for public domain use.

### Sequence analysis

Sequence Ind_*Pf*PMT, *Plasmodium* orthologues worldwide, other organisms and phosphatidylcholine ethanolamine *N-*methyltransferase (PtdEPMT) isoforms (*BRENDA*, EC no. 2.1.1.17) were aligned and using Multalin online server **(**http://multalin.toulouse.inra.fr/multalin/)[20].Plasmodium orthologous proteins of Ind_*Pf*PMT and other species were retrieved From KEGG (http://www.genome.jp/kegg/) and EuPathDB (https://eupathdb.org/eupathdb/). Phylogenetic tree was constructed based on the neighbor joining method using MEGA6.0, at bootstrap confidence replication value 10,000 [21, 22]. Domains of all *Plasmodium* strains were compared using InterPro (http://www.ebi.ac.uk/interpro/) [23–24].

### Inhibitor identification of *Pf*PMT

#### Active site analysis and compound library enumeration

No mutation was found in amino acid sequences of Ind_*Pf*PMT (India) and *Pf*PMT 3D7 (PDB Id: 3UJ9), thus the crystal structure (PDB Id: 3UJ9) was used docking of compounds [25]. The potential active sites were analyzed from SiteMap with default settings applying OPLS_2005 force field where hydrophobicity kept more restrictive, which identified the cavities and scores them according to the site volume and site score. The active site Grid was generated using Glide module of Schrodinger *v9*.*6* to screen the compounds [26–27]. Asinex database was browsed to develop the compound library.

#### Pre filtering, virtual screening and *In-vitro* analysis

Prior, to the virtual screening the compound library was filtered for the Druglikeness predictions. ADMET properties of the drug should be optimum to be absorbed and reached to the site of action. ADMET and toxicity (carcinogenicity, mutagenicity, Cyp2d6 prediction, and toxicity)was predicted using TOPKAT modules of discovery Studio *v 3*.*5* (*DS v3*.5) [28]to remove the compounds with unfavorable ADMET properties at earlier stages [29–30] and were also analyzed forLipinski’ rule [31]. TOPKAT module uses quantitative structural toxicity relationship and similarity searching of the model’s database to identify the toxic properties of compounds as a query compound [32].

For Virtual Screening (VS), workflow of Schrodinger *v9*.*6* was used for screening of compound library in three steps where the dataset size goes smaller at each stage [27]. The pEth/pCholine, endogenous substrate/product were used as docking control. Compounds with better XP Glide score than docking control were selected and binding energy was predicted using Prime MM-GBSA module of Schrodinger *v9*.*6* applying OPLS_2005 force field [33].

For *in-vitro* analysis the *Plasmodium falciparum* parasite culture was maintained in RPMI1640 media supplemented with 5% sodium bicarbonate, 10% heat inactivated human serum. Ring stage parasitized cells were separated from 5% sorbitol. 1% of ring stage parasitized cells were maintained for *in-vitro* analysis of identified compound. The *in-vitro* antimalarial activity assay was performed [34] in 96 well-microtitre plates. The stock solution of each compound (250 μM) was prepared in 0.1% DMSO and the further serial dilutions of each compound were prepared.100 μl of each compound was pipette down in test wells in triplicate and control wells (without test compound) were incubated at 37°C in CO_2_ incubator. Blood Giemsa stained smear was prepared from control and microscopic was done for schizonts formation (1%) after 36–40 h of incubation. The % parasitemia was determined by counting a total of 200 asexual parasites (both live and alive) microscopically using chloroquine as the reference drug. The compounds with IC_50_ less than 5.0 μM were considered as primary hits.

#### Gametocidal and cytotoxicity activity

Gametes of *Plasmodium falciparum* RKL-9 strain were developed from synchronized ring stage of RKL-9 strain using hypoxanthine supplemented complete RPMI1640 mediafor gametocidal activity testing of primary hits. Hypoxanthine necessarily supplements media with purine which is important for development as well as maturation of gametocytes. Stock solutions of primary hits and standard drug primaquine (Sigma-Aldrich) were prepared. On 5–6 day gametocytes were produced and examined morphologically through the microscopy. The plate with 2–3% gametes were incubated at 37°C with primary hits in serial dilution for 48 hrs. Culture without test compound (primary hits) was kept as control. Thin smears were prepared and stained with 10% Giemsa and microscopy was done to examine the next stages of gametocyte (stages II and III). Activity at each concentration of primary hits was calculated in terms of % inhibition [35].

Human embryonic kidney cells 293 **(**HEK293) were developed using Dulbecco’s Modified Eagle’s Medium high glucose medium added with penicillin (1%) and supplemented with fetal bovine serum (5%) and placed in CO_2_ incubator at 37°C for carrying MTT (3-[4,5- dimethylthiazol-2-yl]-2,5-diphenyltetrazolium bromide)toxicity assaybased on colorimetric assay.Before seeding five thousand cells into 96-well plate, were washed thrice with growth media and trypsinized. Cells with viability 98% were added with primary hits in different dilutions (10μM, 50μM, 100μM, 150μM, 200μM, 250μM) to the plate and incubated for 24 hours. After 24 hrs incubation MTT 5mg/ml was added to the plate and 4 hrs incubation was given. The supernatant was removed and added with DMSO to each well and the readings of absorbance were recorded at 580nm using Synergy/HTX MultiScan reader (BioTek) [36–37].The Lethal dose LD_50_ of each primary hits were calculated as mean from experiments in triplicate. Based on the LD_50_ and antimalarial IC_50_ of each inhibitor were used for selectivity index (SI) calculation.

#### *Pf*PMT expression, purification and enzymatic inhibition assay

*Pf*PMT cDNA was synthesized and cloned using pET-24a expression vector. Positive pET24a clone was then transformed in BL21 (DE3) expression host for protein expression and purified. In LB medium expression strains were incubated at 37°C and added with Kanamycin (Kanamycin: 35mg/ml stock concentration).The culture was grown to an OD of 0.6 at 600 nm and induced with 1mM isopropyl-β-D- thiogalactopyranosidase (IPTG).Before and after induction samples were analyzed on SDS-PAGE (12%). The culture was pelleted down and suspended in 1X PBS. 1X PBS suspended pellet was subjected to sonication and was centrifuged after sonication at 10,000 rpm for 15 min at 4°C. Sonication supernatant and pellet were loaded on 12% SDS-PAGE for checking the protein of interest. SDS-PAGE analysis of 1X PBS sonication showed the presence of protein of interest in supernatant. The supernatant was further processed for protein purification using gel extraction protocol. Purified fractions were loaded on 12% SDS-PAGE and further confirmed by MALDI TOF MS/MS analysis.

Native form of *Pf*PMT was confirmed through assay which was performed in UV-transparent plates (acrylic, nonsterile, costar) using *SAM-dependent methyltransferase assay Kit of G-Biosciences* which works on the degradation of Adenosylhomocysteine (SAH) into urate and hydrogen peroxide by mixture of enzymes. 1mM SAH was used as positive control. The assay mixture was consisted with SAM methyltransferase assay buffer with phosphoethanolamine (pEth) 200μM, SAM 200μM, 2.5μM *Pf*PMT, SAM enzyme mixture and SAM colorimetric mix in a total volume of 115μM. The functional concentration of phosphoethanolamine was confirmed according to the *Pf*PMT assay developed by Bobenchik et al [38]. Finally, the rate of formation of hydrogen peroxide was measured by increase in absorbance at 510 nm.

The Primary hits were dissolved in 1% DMSO solution and stock solution was prepared in H_2_O. The experiments of all primary hits were carried out in presence of native purified *Pf*PMT protein and the absorbance was quantified at 510 nm. The absorbance of protein without inhibitors was considered as 100% protein activity. The absorbance at different concentrations of each primary hits were converted into % *Pf*PMT inhibition and plotted against inhibitor concentration. Enzymatic assay was also performed at different increasing concentration of substrate pEth (50, 100, 150, 200, 250 μM) to determine the Ki value for enzyme kinetics.

#### *In vivo* studies

Animal experiment studies were carried out at JALMA, Agra, India with the grant of ethical approval from the Animal Research Ethics Committee. Compounds were procured from Asinex database with 98% HPLC purity and the analytical grade chemicals and reagents were used in the study. Triple distilled Millipore purified water (Millipore, USA) were used to prepare solutions. Male albino mice of twelve to sixteen months old, weight ranging 22 to 25 g used in study. Mice were exposed to the day/night light cycle of 12 hours period. Mice were monitored properly with plentiful dried food and water and their sanitation was carefully monitored every day (S1 Protocol).

The parasite *Plasmodium berghei* (strain ANKA) is cryopreserved and stored at parasite bank ICMR-National Institute of Malaria Research, New Delhi. Two donor mice were infected with *Plasmodium berghei* parasite, administered intraperitoneally one week before the date of experiment to be conducted. The infected blood from donor mice was collected from tail bleeding method into heparinized tubes and parasitemia (≥15) was determined using microscopy. Mice were divided into three groups as test group, Positive control group and negative control group. Three mice for each group were used and each mouse was administered calculated volume of saline water (0.9% NaCl). 200μl of *P*. *berghei* infected RBCs (1×10^7^ per 200μl) were given to each test mice to infect them. Dosages were calculated as per body weight and route of administration orally (gavage needle) and Intravenous (IV) and standard dose selected. Mice group with parasitized erythrocytes kept a positive control. One group of three mice were treated with chloroquine (CQ) as negative control and the test mice were administered with studied *Pf*PMT inhibitors (test compounds) once a day. The first dose was administered on first day two hours later of the infection followed by other three doses each at 24 hours interval. On fifth day the blood sample was collected from tail bleeding, collected in tubes containing PBS and % parasitemia was checked using microscopy and % growth inhibition was calculated [39]. The mice were scarified through using CO_2_ to terminate the experiment [40].

## Results and discussion

### *Pf*PMT gene amplification and sequence analysis

Analysis of sequenced Ind_*Pf*PMT gene using FINCHTV showed that gene is carrying open reading frame of 801 bp coding nucleotide and Blastn analysis showed the 100% nucleotide identity with *Pf*PMT*3D7* implied the functional and structural conservation at gene as well as protein level too. Amino acid sequence analysis of Ind_*Pf*PMT and its orthologues was done through the multiple sequence alignment (MSA) and Phylogenetic analysis. Since, *phosphoethanolamine methytransferase* (PMT) gene is absent in human, so the Blastp of Ind_*Pf*PMT was done with human proteome and protein with sequence identity >20% was selected for sequence analysis. Human protein PtdEPMT isoforms (Table 1) with identity of 27% were used for analysis. The MSA showed 100% amino acid sequence identity with *Pf*PMT*3D7* and 66% with *Pv*PMT isolates worldwide. Orthologues (*Pk*PMT, *Pr*PMT, *Pc*PMT) also showed significant identity and sequence coverage with Ind_*Pf*PMT.

**Table 1:** Depicted domain and motifs scan of PMT proteins of *Plasmodium* species.

Plasmodium Strain	No. of amino acids	Domain (SAM dependent methyltransferase)	Methyltransferase Motifs
*PF*PMT_Indian	266	(aa19 –aa265)	aa 60–157
*PF*PMT3D7_1343000	266	(aa19 –aa265)	aa 60–157
*Pr*pmt_A0A060RY81_PLARE	266	(aa19 –aa265)	aa60-157
*PV*PMT Sal1	264	(aa18 –aa262)	aa57-154
*PV*PMT_India	264	(aa18 –aa262)	aa57-154
*PV*PMT_Brazil	264	(aa18 –aa262)	aa57-154
*Pk*NH_B3L8G9	264	(aa24 –aa262)	aa57-154
*PV*PMT_Mauritania I	264	(aa18 –aa262)	aa57-154
*PV*PMT_North Korean	264	(aa18 –aa262)	aa 57–154
PMT_P.inui San Antonio 1	274	(aa34—aa272)	aa67-164
*PC*PMT.strainB_K6UVQ5	258	(aa25 -aa 256)	aa51-148

**Fig 1:**
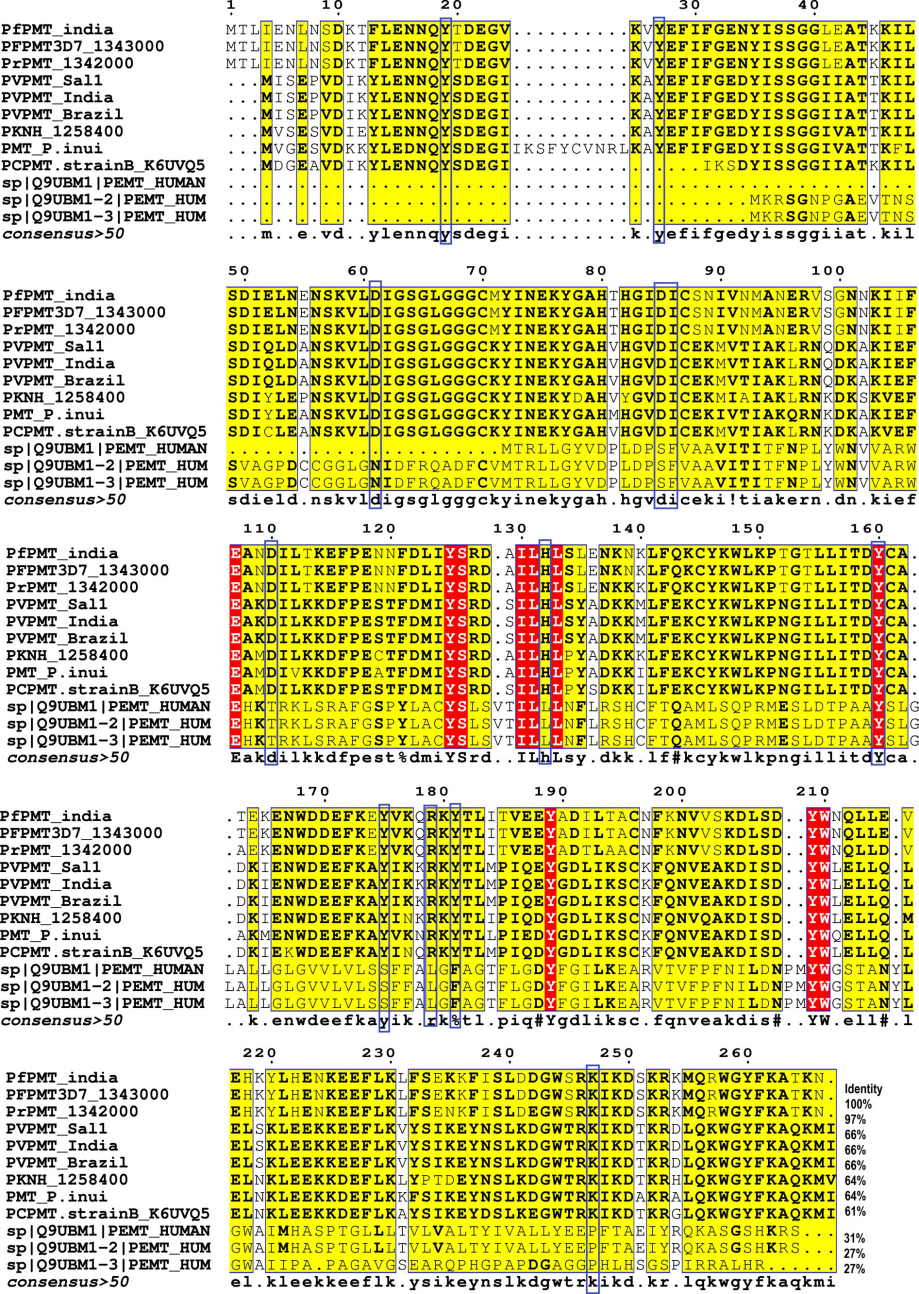
Multiple alignments of Ind_*Pf*PMTare depicted with its orthologs from *Plasmodium* and human PEMT (Phosphatidylcholine ethanolamine methyltransferase) enzyme isoforms. Crucial amino acids for biological function of *Pf*PMTare shown in blue boxes.

The amino acids in the red background were found conserved both in *plasmodium* PMT and human phosphatidylcholine ethanolamine *N*-methyltransferase(PtdEPMT) isoforms. Besides the 27% of identity of *Pf*PMT with PtdEPMT, none of the crucial residues except Tyr160 were found identical that implied the big difference within the binding pocket of both the proteins. Tyrosine residues Tyr19, Tyr-27, Tyr-160, Tyr-175, and Tyr-181 enclosed in blue boxes which are crucial for phosphoethanolamine/phosphocholine binding for biological function were found conserved throughout the *plasmodium* PMT orthologues. The arginine Arg179 and Lysine Lys247responsible for electrostatic interaction within the phophobase site were also found conserved. The residues Tyr19 and His132 essentialfor forming catalytic dyad between AdoMet and phosphobase were also found conserved among *Plasmodium* spps globally (Fig 1). Hence, Ind_*Pf*PMT has excellent functional and structural conservation among demographic isolates of PMT protein orthologues that makes it a potential therapeutic target for structure based drug discovery. The entirely different binding pocket from PtdEPMT may be helpful in developing the *Pf*PMT specific inhibitors without side effects to human.

**Fig 2:**
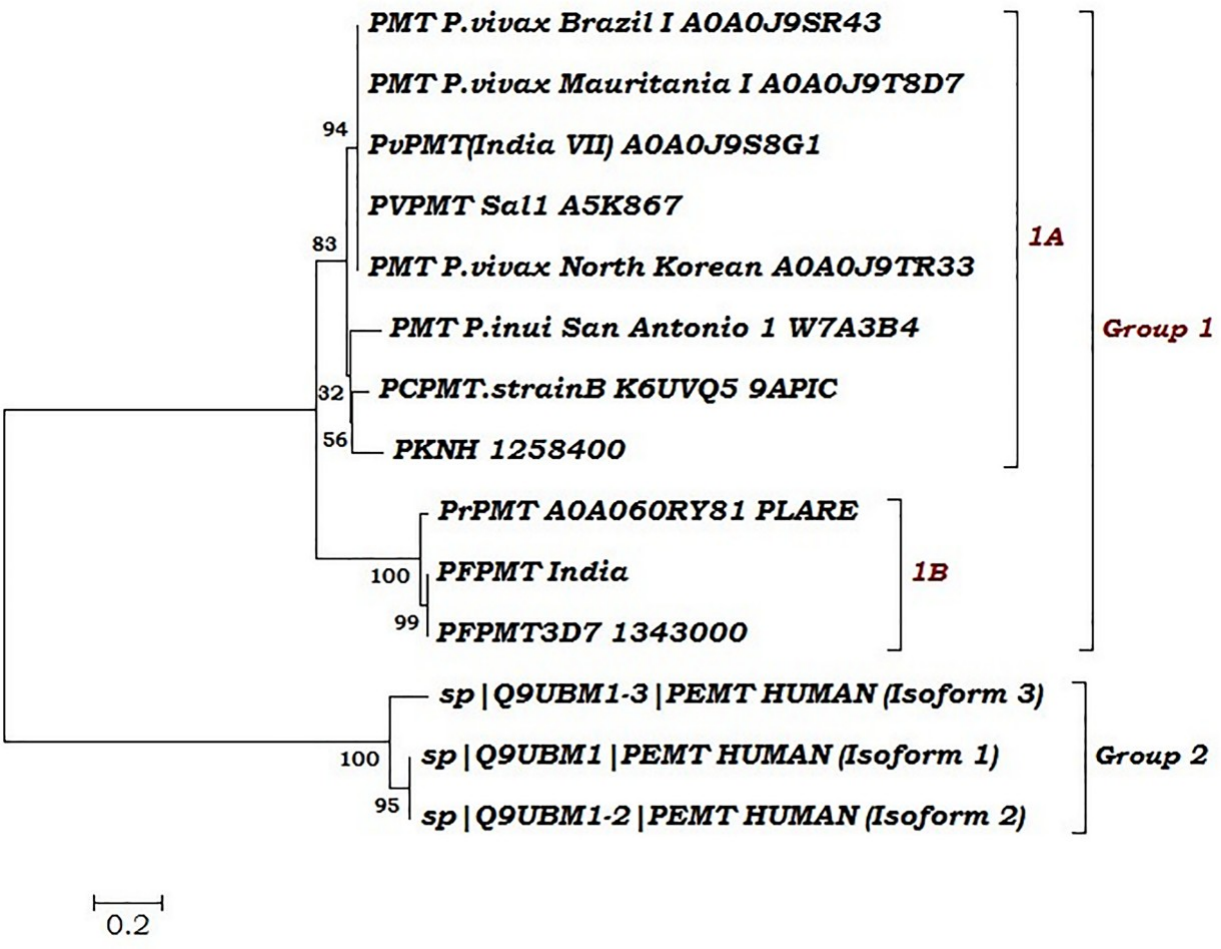
Phylogenetic analysis of Ind_*Pf*PMT is shown with other pmt orthologues in *Plasmodium* species using neighbor joining method.

Phylogeny was studied separately to better understand the evolutionary relationship and global distribution of PMT protein among orthologues of *Plasmodium* worldwide and other spp. Phylogenetic tree revealed that all studied PMT protein members clustered into two major groups **(**Fig 2**)**. Group 1 formed cluster of PMT protein of all *Plasmodium* spp. isolates globally and group 2 is a unique distant cluster of human phosphatidylcholine ethanolamine *N-*methyltransferase (PtdEPMT) isoforms. Group 1 was subdivided into two related clustered protein i.e. 1A and 1B. In subgroup 1A, human malaria parasite PMT of all *Plasmodium vivax*, *P*. *knowlesi*and nonhuman malaria parasite *P*. *cynomolgi* isolates formed a unique cluster. Ind_*Pf*PMT was clustered with *Pf*PMT3D7 and *P*. *reichenowi* in a group 1B. Phylogeny implied the close evolutionary relatedness between ind_*Pf*PMT and Plasmodium PMT orthologues and a very distant relation with human PtdEPMT isofoms with very less sequence identity (Fig 2).

**Fig 3:**
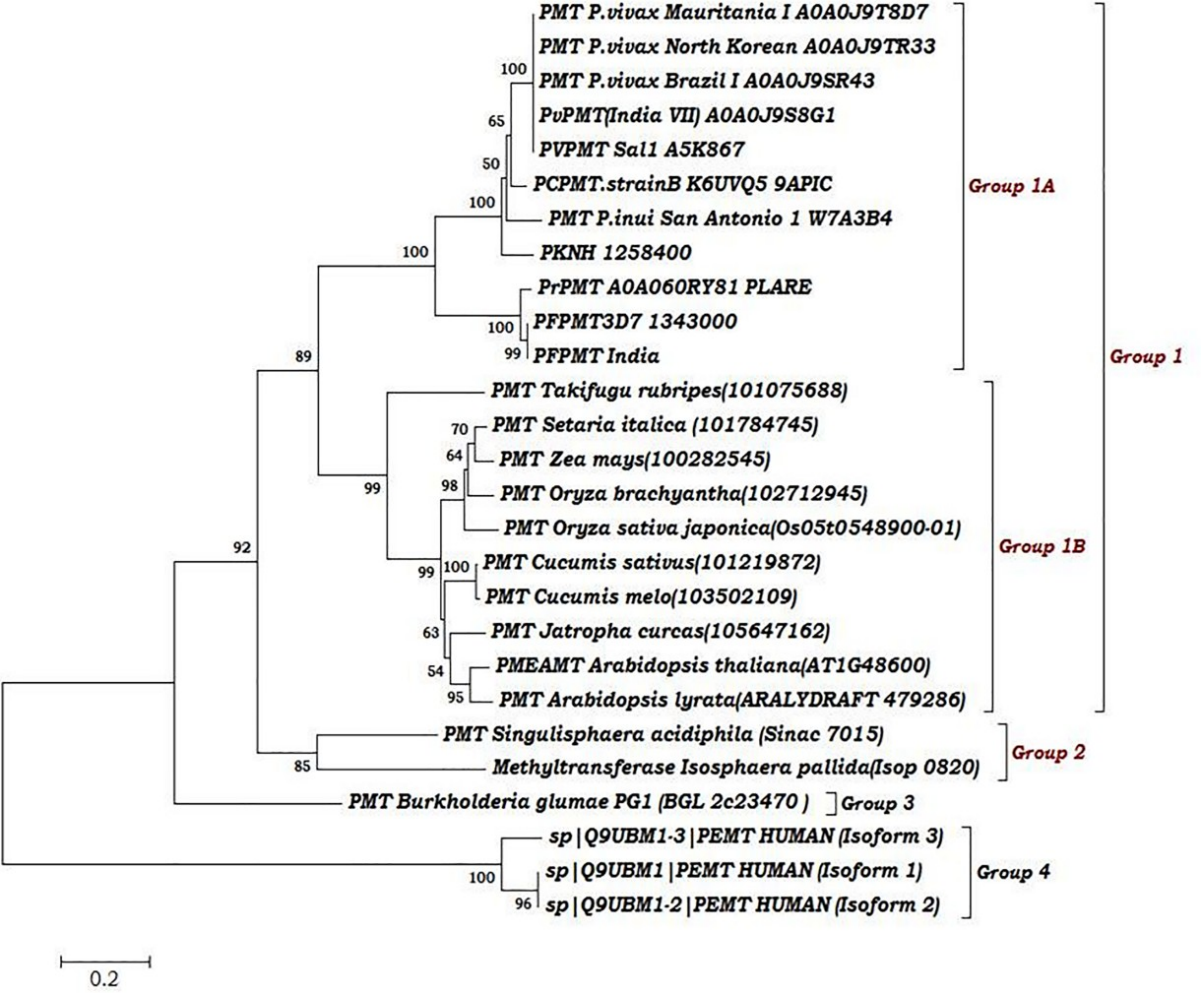
Phylogeny showed with all *Plasmodium* and other species orthologues from KEGG and EuPathDB along with human PtdEpmt.

Phylogenetic analysis of PMT of Plasmodium with orthologues from other spp showed the peculiar distribution of PMT gene among different protozoan pathogens and plants. PMT gene has common ancestor to all the apicomplexans of group 1A and group 1B that revealed the close evolutionally relationship among PMT orthologues, fungi (group 2) and plant orthologues (group 1B). Humans PtdEPMT formed distant separate group (group 4) **(**Fig 3**)** which also revealed the distant relation with human PtdEPMT with negligible functional as well as structural conservation so, drug developed based upon Ind_*Pf*PMT may be effective against different human *Plasmodium*species globally without any side effects.

Domain is a super secondary structural part called as catalytic unit of a protein. A protein can have multiple domains or whole protein can act as single domain. Domains can be comprised of different secondary structural motifs responsible for catalyzing specific function. Secondary structural analysis (Table 1) revealed that Ind_*Pf*PMT and *Pf*PMT3D7 were found to have 100% conserved methyltransferase motif and SAM dependent methyltransferase domain with significant identity with *Plasmodium* PMT orthologues which makes it an important drug target for structure based drug discovery **(**Fig 1**)**. It has also been found that *Pf*PMT3D7, *Pv*PMT and *Pk*PMT have significant sequence and structural similarity [17]. Hence, Ind_*Pf*PMT may fulfill the thrust of potential drug effective globally based on structure based computational drug development, which may be effective against human infecting *Plasmodium* isolates worldwide.

### Inhibitor identification of *Pf*PMT

#### Protein structure and compound library for docking

Since no mutation was found among Ind_*Pf*PMT (Indian) isolate and reference gene (*Pf*PMT*3D7*), Ind_*Pf*PMT and *Pf*PMT3D7 are structurally and functionally 100% identical. SAM bounded of *Pv*PMT and *Pk*PMT structures have shown hydrophobic PMT active site and having very similar structural folds with RMSD = 0.452 A°. The well conserved active sites of *P*. *falciparum*, *P*. *vivax* and *P*. *knowlesi* could potentially be inhibited by a common inhibitor which may act as an effective antimalarial drug globally [17].Hence, the crystal structure of *Pf*PMT*3D7* (PDB Id: 3UJ9) was used for virtual screening of compound library. Compound library was built from Asinex compound library.

#### Active site creation

Binding sites of (pCholine or pEth) and (AdoMet or AdoCys) are adjacent to each other and there catalytic dyad is formed between Tyr19 and His132 from pCholine/pEth and AdoMet/AdoCys binding sites respectively [25]. Active sites scored from SiteMap, the potential active site having site score 1.059 and drugscore 1.004 found with the bigger site volume (716.87). The important thing found with this site is the pCholine/pEth and AdoMet/AdoCys binding pockets are covered by the site. Grid was generated around this site and increased upto 12A^o^ in XYZ direction so that compound can move easily within the pocket and can generate ideal conformation.

#### Pre filtering, virtual screening and *invitro* analysis

Since compounds which fulfill the parameters of Lipinski rule have better probability of druglikeness, so prefiltering of compounds before docking is a good approach. Compound library was pre filtered through Lipinski rule of five and ADMET using *DSv3*.5 and promiscuous compounds using *false positive remover* to save time and experimental cost. False positive remover identifies and eliminates the compounds with reactive functional groups like compounds containing sulfonamide, rhodanine, quinine, benzpyrenone and catechol as these promiscuous compounds have the probability of binding covalently nonspecifically within active pocket. According to the physicochemical parameters, a drug with high solubility may not able to cross membranes and highly lipophilic drug have dissolving problem. So, there should be optimum ratio between lipophilicity and water solubility according to the Lipinski rule of five. Therefore, the curated non-promiscuous compound with better Lipinki rules’5 were subjected for computational ADMET analysis. Since solubility plays significant role from dissolution to the drug action and according to the *DS v3*.5, for a druggable compound solubility must be ranging from extremely low solubility (−8.0) to an optimal solubility (0.0). Solubility levels 3 (good solubility) and 4 (optimal solubility) signified that selected five compounds have druggable solubility as given in Table 2. According to the *DS v3*.5, the lipophilicity (ALogp98) value should range between the −2.0 and 5.0 and PSA should not be more than 140Å for good intestinal absorption. Absorption level 0 and 1 suggested the good and moderate absorption probability respectively, which fulfill the druglikenss filters of cell permeability and may be absorbed in the intestine. On computational toxicity analysis, selected inhibitors of *Pf*PMT showed no affinity for Cyp2d6 (Cytochrome enzyme) and plasma proteins. Thus, these may not be responsible for any kind of drug- drug interaction and retention of these compounds within the human body. These were also passed through the toxicity filter and found to have nontoxic properties as given in Table 2.

**Table 2:** Docking score and binding energy of selected compounds tabulated with interacting amino acids and Virtual ADME parameters as per Lipinski rule of five of selected compounds and virtual toxic physicochemical parameter analysis of selected compounds.

Compound ID	ASN.1	ASN.2	ASN.3
IUPAC name	6-[Amino-2-(4-fluoro-phenyl)-ethyl]-pyridin-2-yl-1*H*-pyrimidin-4-one	1-{2-[(5-Phenyl-furan-2-ylmethyl)-amino]-ethylamino}-propan-2-ol	*N*-[2-(3,4-Dimethoxy-phenyl)-ethyl]-2-[(tetrahydro-furan-2-ylmethyl)-amino]- acetamide
Mol. weight	311.3	308.8	323.4
H_Acceptor	5	4	6
H_Donor	4	3	3
PSA	84.9	58.9	56.9
LogP	0.40	2.46	-0.15
ROS Violation	0	0	0
Solubility	-2.3	-3.0	-0.84
Solubility level	3	3	4
Absorption level	0	0	0
CyP2D6 affinity	False	False	False
Mutagenic	False	False	False
Carcinogenic	False	False	False
Tumergenic	False	False	False
**Glide XP score**	**-12.3**	**-9.81**	**-9.66**
**Binding energy**	**-110.2**	**-98.4**	**-96.8**
**IC**_**50**_ **(Schizonticidal)**	**1.77μM**	**3.93μM**	**3.21μM**

Hence, selected compounds were found to have well to optimum drug likeness properties and also found non-carcinogen, non-mutagen, and nontoxic were subjected for docking analysis using Schrodinger *v9*.*6* through three consecutive docking stages based on precision and accuracy. The Asinex compound library was subjected for docking analysis based on precision and accuracy.

The pCholine and pEth (docking control) were docked with *Pf*PMT and found score -5.6 kcal/mol and -7.2 kcal/mol glideXP respectively and the Prime MMGB binding energy was calculated as -49.8 kcal/mol and—54.4 kcal/mol respectively. To minimize the computational error compounds with Glide XP score more than Glide XP cutoff ≤ -10 kcal/mol was considered as better compounds and further validated as per binding energy (Prime MMGB) of docking compounds. Compounds with binding energy cutoff ≤ -60kcal/mol, better than docking control were selected as hits and were found preferably thermodynamically stable conformation with lowest possible energy within the binding.

Three hundred hits were found binding with crucial amino acids (Tyr amino acids) and amino acids (Tyr19 & His132) crucial for catalytic dyad within the *Pf*PMT binding pocket were selected and clustered for selecting diverse chemotypes to test and different promising chemotypes. Clustering was done based on tanimoto coefficient similarity index using Canvas, Schrodinger *v9*.6. Hits were clustered into seven groups based on tanimoto groups containing chemotypes (Phenyl-tetrazolo-pyridin, Pyrrolo-pyridinyl, pyridinyl pyrimidine, furanyl- piperidine, morphaline, Benzoimidazol, phenoxychromen, phenyl- furan, etc.) and top scored druglike compound representative from each group were procured for in vitro analysis.

**Fig 4:**
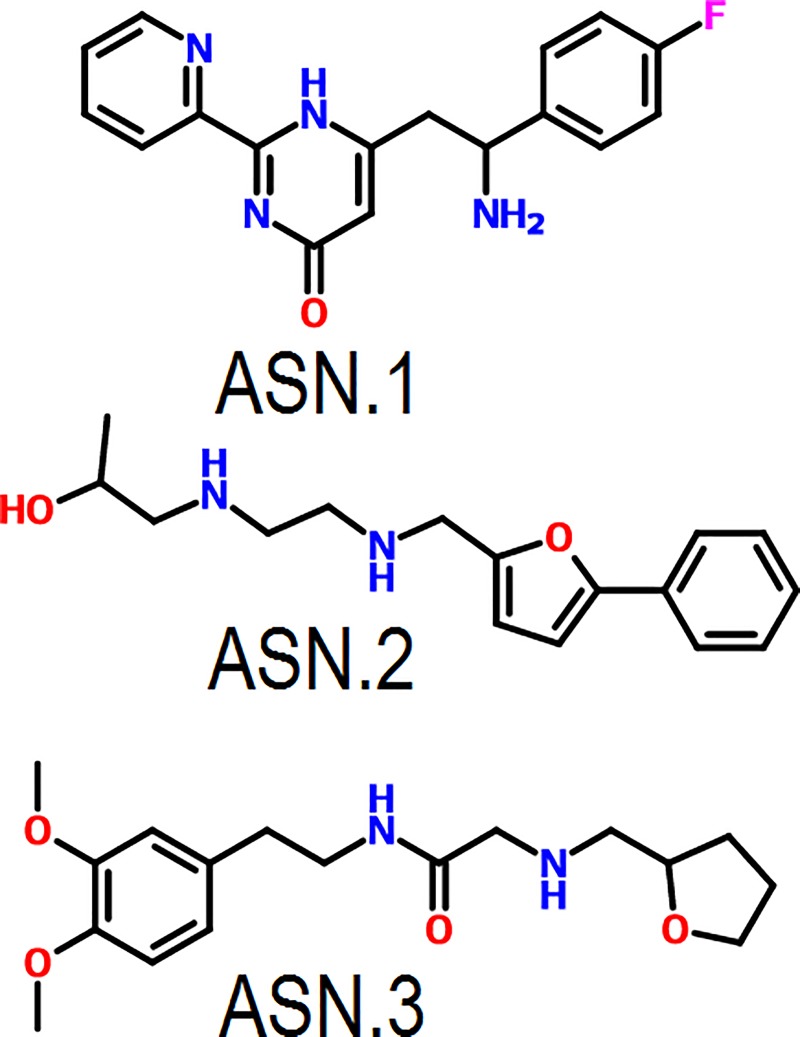
Structures of the three primary hits which showed good schizonticidal activity.

All procured compounds were tested on parasite culture in triplicate and only three compounds (Fig 4) showed good schizonticidal activities on *Plasmodium falciparum* culture. IC_50_ was calculated based on parasite inhibition at different concentrations and compounds with IC_50_< 5μM were selected as primary hits for further analysis. Compounds formed binding with essential amino acids.

**Fig 5:**
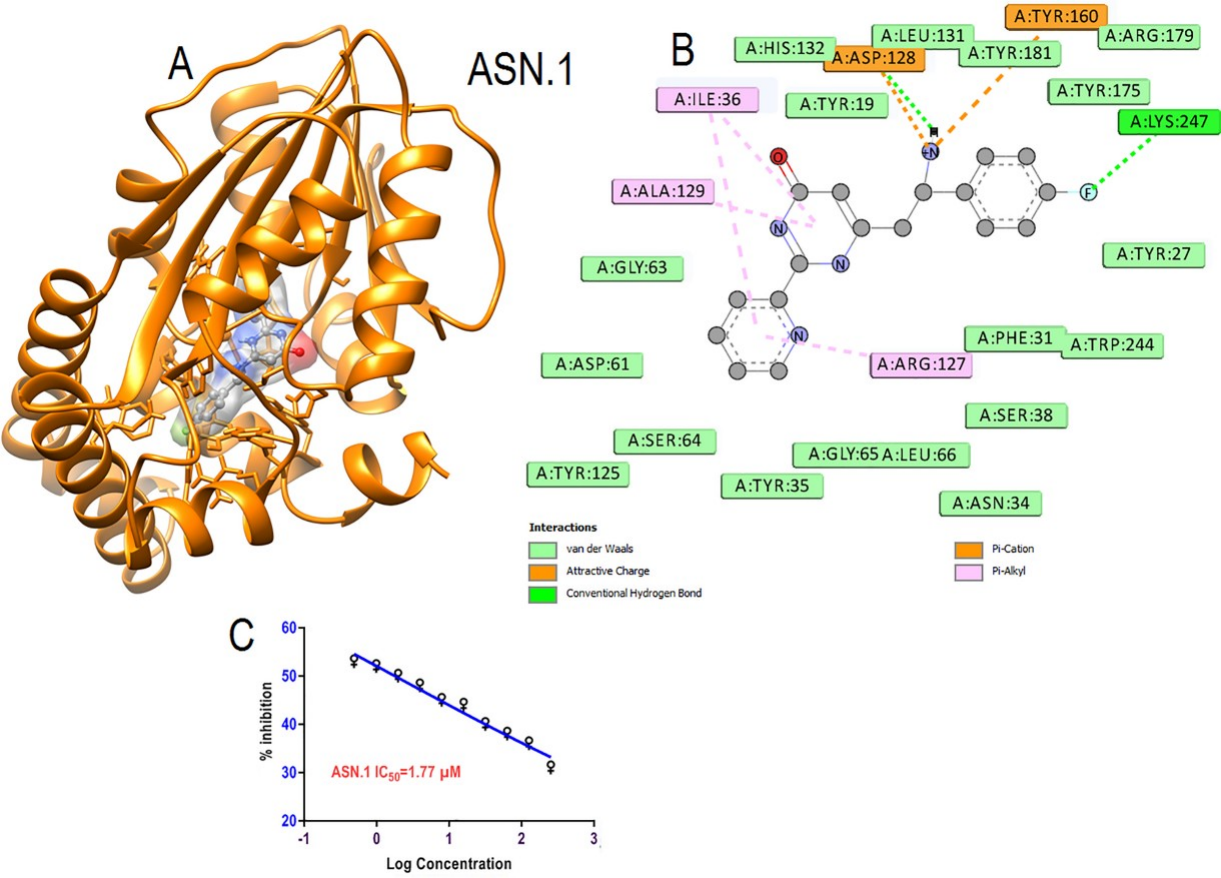
**(A)** Interaction of primary hits (ASN.1) is shown in element color ball and stick in surface view within the active pocket of *Pf*PMT. **(B)** 2D interaction with amino acids within five Å of interaction.**(C)** Schizonticidal activity (IC_50_)against growth of *Plasmodium falciparum* parasite culture.

The primary hit ASN.1 occupied the substrate pocket, shown in surface view (Fig 5A). ASN.1 fluorine of benzene ring formed H-bonds with Lys 247 crucial for binding of substrate. Electrostatic interaction were formed with Tyr 160 and Asp 128 through π-cation bonding and also formed vanderwaal interactions with important tyrosine residues like Tyr 19, Tyr 175, Tyr 181 and other residues like His 132 and Arg 179 essential for biological function of *Pf*PMT for substrate binding and catalytic dyad formation. Both the heterocyclic ring (pyridine and pyrimidine) packed performing hydrophobic interaction throughπ-alkyl bondinginto binding pocket (Fig 5B). Since, hydrophobic interactions are as crucial as hydrogen interaction so, the interaction of ASN.1 with crucial residues within binding pocket implied significance of interactions for schizonticidal activity at very low μM concentration IC_50_ at 1.77μM (Fig 5C).

**Fig 6:**
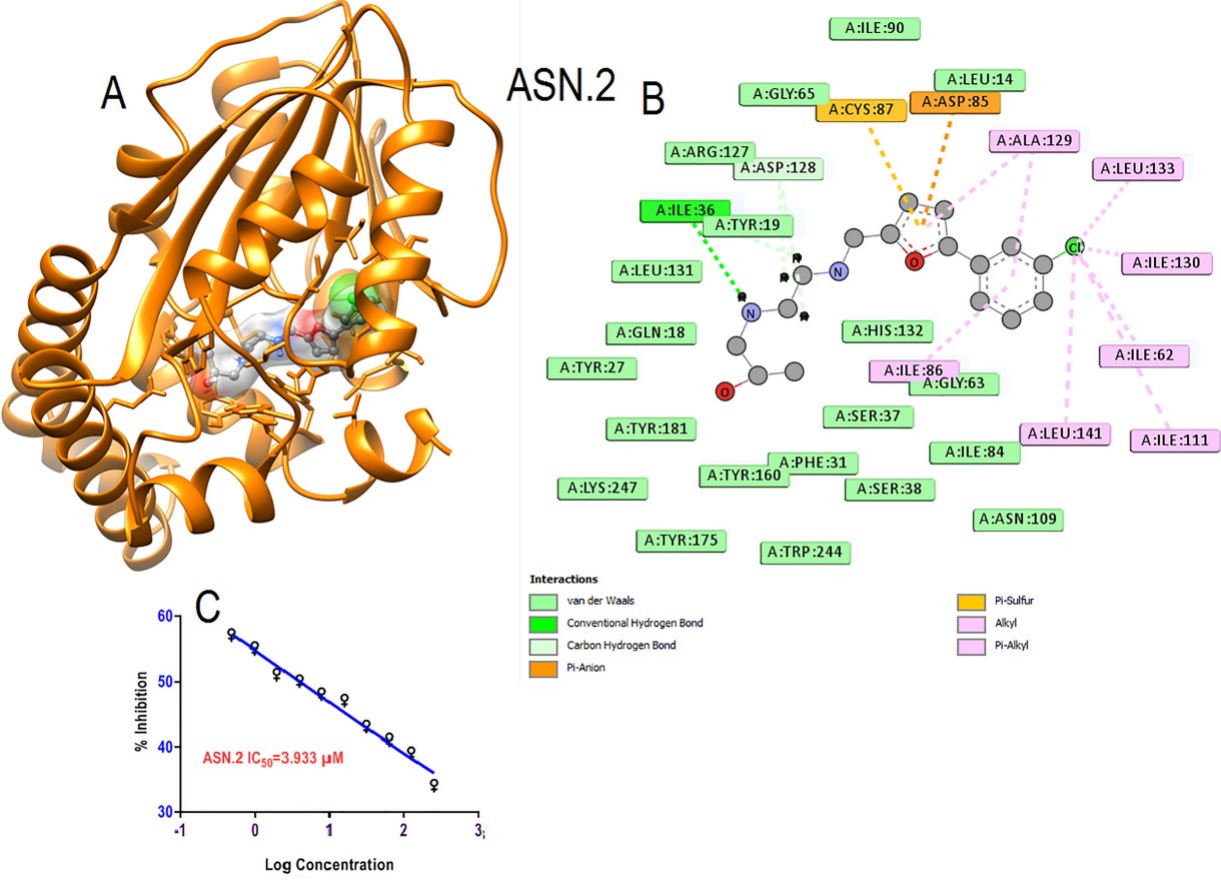
**(A)** Interaction of primary hits (ASN.2) is shown in element color ball and stick in surface view within the active pocket of *Pf*PMT. **(B)** 2D interaction with amino acids within five Å of interaction.**(C)** Schizonticidal activity (IC_50_)against *Plasmodium falciparum* growth.

The Primary hit ASN.2 found to be occupied within binding pocket of both the substrate and co substrate, SAM (Fig 6A). In ASN.2 chlorine of the benzene ring and furan provided hydrophobic interaction through the alky and π-alkyl bonds with leucine 133, 141 and isoleucine 62, 111 and 130 amino acids within the hydrophobic core of binding pocket.Vander wall interaction formed with tyrosine residues like tyr19, 160, 175 and Tyr 181 and other amino acids His132, Lys 247, Arg 179responsible for substrate binding as well as catalytic dyad formation (Fig 6B). Furan ring also showed π- anion interaction and hydrophobic interaction throughπ-alkyl bonding with inhibitor. Interactions showed may prevent the formation of catalytic dyad and substrate binding which may be the reason of good inhibition of schizonts at low micromolar concentration (IC_50_ = 3.93μM)(Fig 6C).

**Fig 7:**
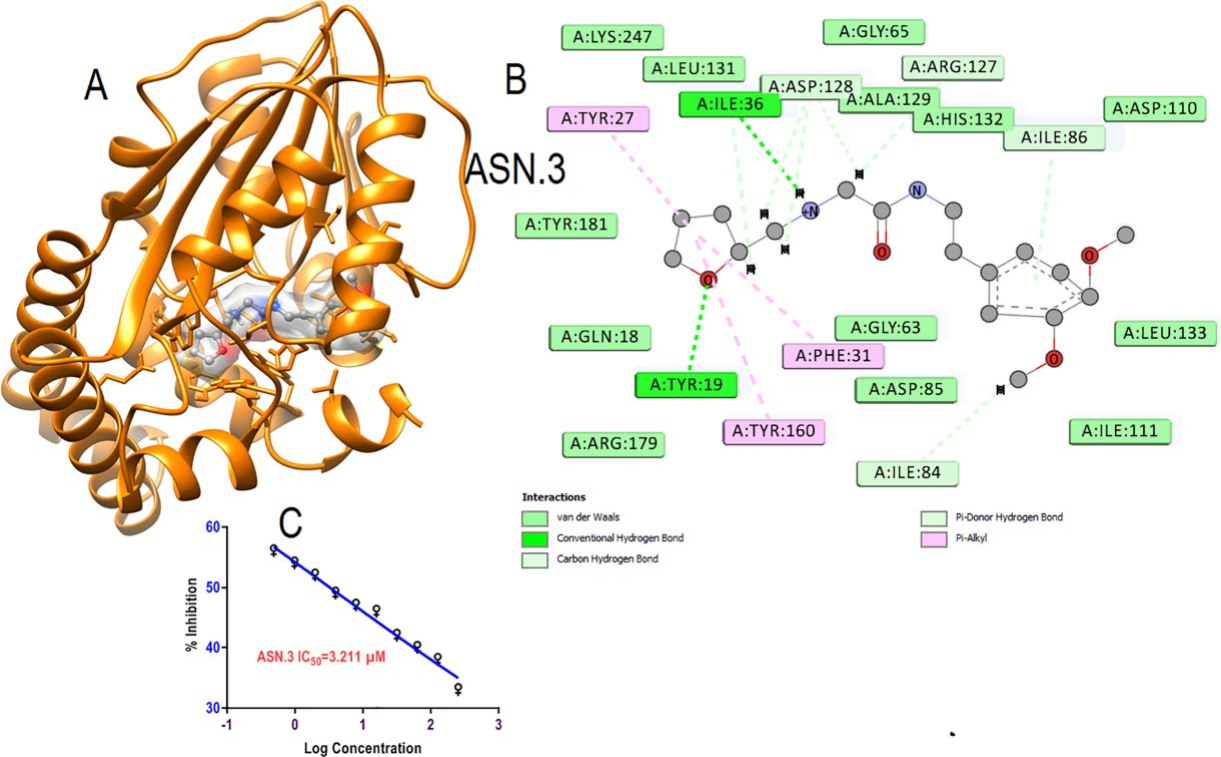
**(A)** Interaction of primary hits (ASN.3) is shown in element color ball and stick in surface view within the active pocket of *Pf*PMT. **(B)** 2D interaction with amino acids within five Å of interaction.**(C)** Schizonticidal activity (IC_50_)against growth of *Plasmodium falciparum*.

Primary hit ASN.3 showed good interaction within the binding pocket by occupying crucial amino acids (Fig 7A).The oxygen of furan heterocyclic ring formed hydrogen bonds with crucial amino acids Tyr19 for binding with substrate for catalyzing the function. Vander wall interaction formed with His132, Tyr 181, Lys 247, Arg 179 and Hydrophobic interaction by furan ring throughπ-alkyl bonding with Tyr 160 crucial amino acids binding of substrate and for catalytic dyad which may be the reason of good inhibition of schizonts at low micromolar concentration (Fig 7B). The good schizonticidal activity at 3.21μM low micromolar concentration may be because of the good interaction within the binding pocket occupied key essential amino acids (Fig 7C).

The docking analysis implied better affinity for the *Pf*PMT that the three compounds interact with crucial amino acids which may prevent the binding of substrate and also the transmethylation of phosphoethanolamine within the binding pocket. Different chemotypes were tested against *P*. *falciparum* culture and showed low to significant inhibitory activity where primary hits containing pyridinyl pyrimidine (ASN.1) and phenyl- furan (ASN.2 & ASN.3) chemotypes as substructure showed good inhibitory activity against *Pf*PMT. Three primary hits showed good interaction with target protein and formed hydrogen bonds with crucial conserved amino acids for transmethylation as well as inhibition (IC_50_< 5μM) on *Plasmodium falciparum* culture (Table 2).

#### Gametocidal activity and cytotoxicity assay

**Fig 8:**
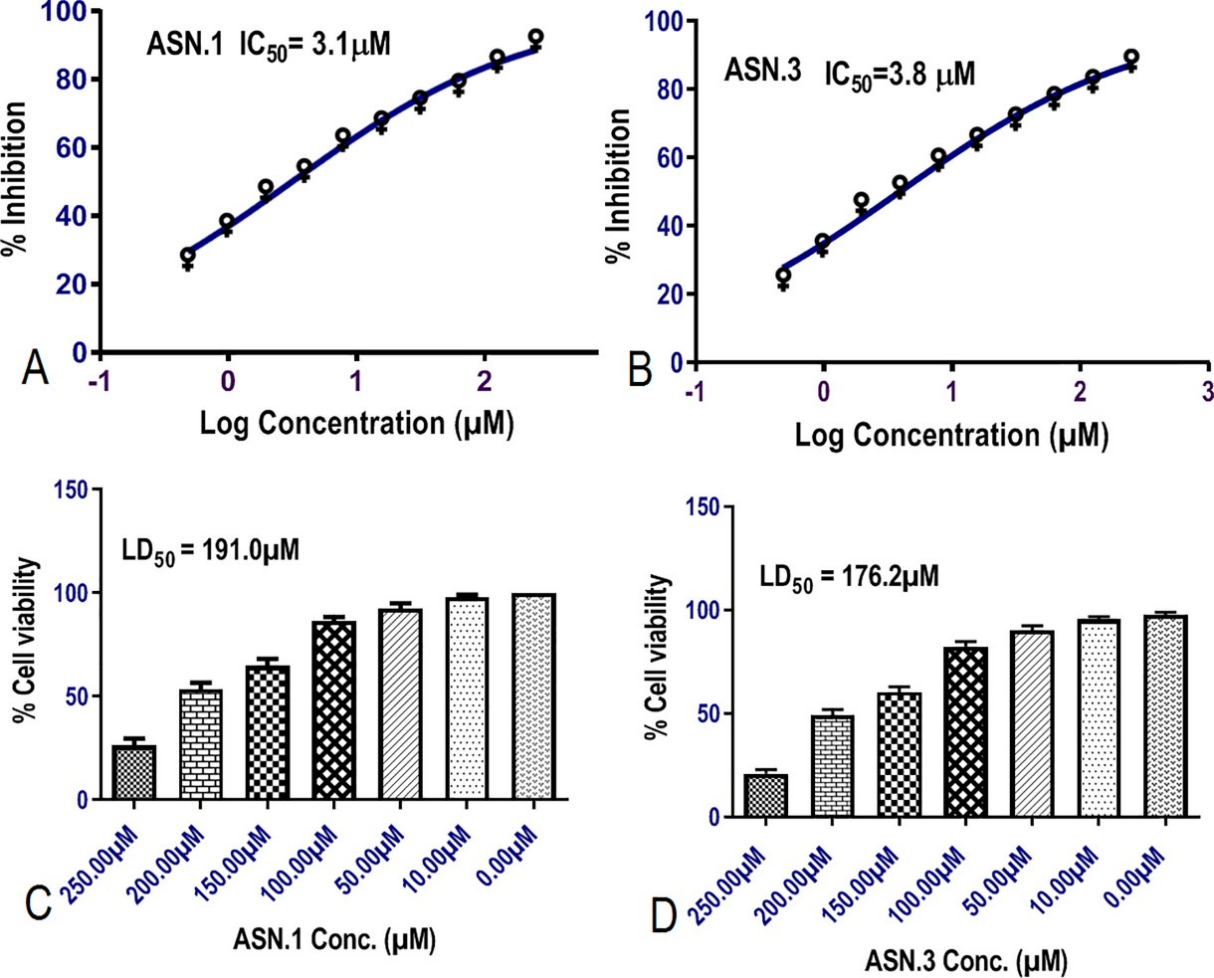
Showed gametocidal activity of primary hits against *RKL-9* strain in terms of IC_50_ **(A, B)**. MTT toxicity of primary hits against HEK-293 cells with P *** (p>0.001) **(C, D)**.

Primary hits were again tested for gametocidal activity and cytotoxicity activity before protein inhibition assay. Primary hits were added in serial dilution to the *RKL-9* gametocytes culture at time of first stage of gametocytes growth and IC_50_ was calculated **(**Fig 8). Primary hits which could inhibit the development of further stages of gametocytes at concentration below 5 μM were kept for further analysis. Primary hit ASN.1 and ASN.3 showed the better gametocyte inhibition with IC_50_ 3.1μM (Fig 8A), and 3.8μM (Fig 8B) respectively. Hence, the significant gametocidal activity at lower micromolar concentration (<5μM) confirmed the dual activity of compounds that these compounds showed the significant activity growth (asexual development stage) and multiplication stage (gametocyte development). Primary hits (ASN.1and ASN.3) were kept for further study and ASN.2 was eliminated (Table 3).

**Table 3:** The activity of inhibitors towards gametocytes, cell cytotoxicity and inhibition kinetics (*Km* and *Ki*) of *Pf*PMT protein.

Compound ID	Gametocidal IC_50_	Cytotoxicity LD_50_	Inhibition EC_50_	*K_m_*	*K_i_*
**ASN.1**	3.1μM	191.0 μM	1.49 μM	52.4 μM	1.2 μμM
**ASN.3**	3.8 μM	176.2 μM	2.31 μM	80.2 μM	2.2 μM

Colorimetric analysis based toxicity of Primary hits was tested for human cell lines using MTT assay against kidney cell line (HEK-293). Primary hits were tested at different concentrations (10μM, 50μM, 100μM, 150μM, 200μM, 250μM) (Fig 8). Five thousand HEK-293 cells were embedded with different concentration of primary hits and fortunately, primary hits ASN.1 and ASN.3 were found nontoxic even at higher concentration with very high LD_50_ concentration 191.0 μM (Fig 8C) and 176.2 μM (Fig 8D) respectively and better selectivity index 1005.26 and 54.8 respectively as per Eq 1. More than 50% of HEK-293 cells were found viable even at 150μM. Even the 90% of cells were viable at 10μM (much higher than the schizonticidal IC_50_), found safer implied that the primary hits had good selectivity index.

$$Selectivity\;Index=LD_{50}/IC_{50}$$ Eq 1

In the *insilico* toxicity analysis, these hits free of any reactive functional group were also found nontoxic (non-carcinogenic, non-mutagenic and non-tumorgenic) Hence, our computational results were found to be correlated with experimental data. The high selectivity index implied the better therapeutic safety of inhibitors and no toxicity entailed the use of these hits as antimalarial. The primary hits with better Gametocidal effect and safety index were taken for further experimental analysis.

#### *PF*PMT expression, purification and enzymaticinhibitionassay

**Fig 9:**
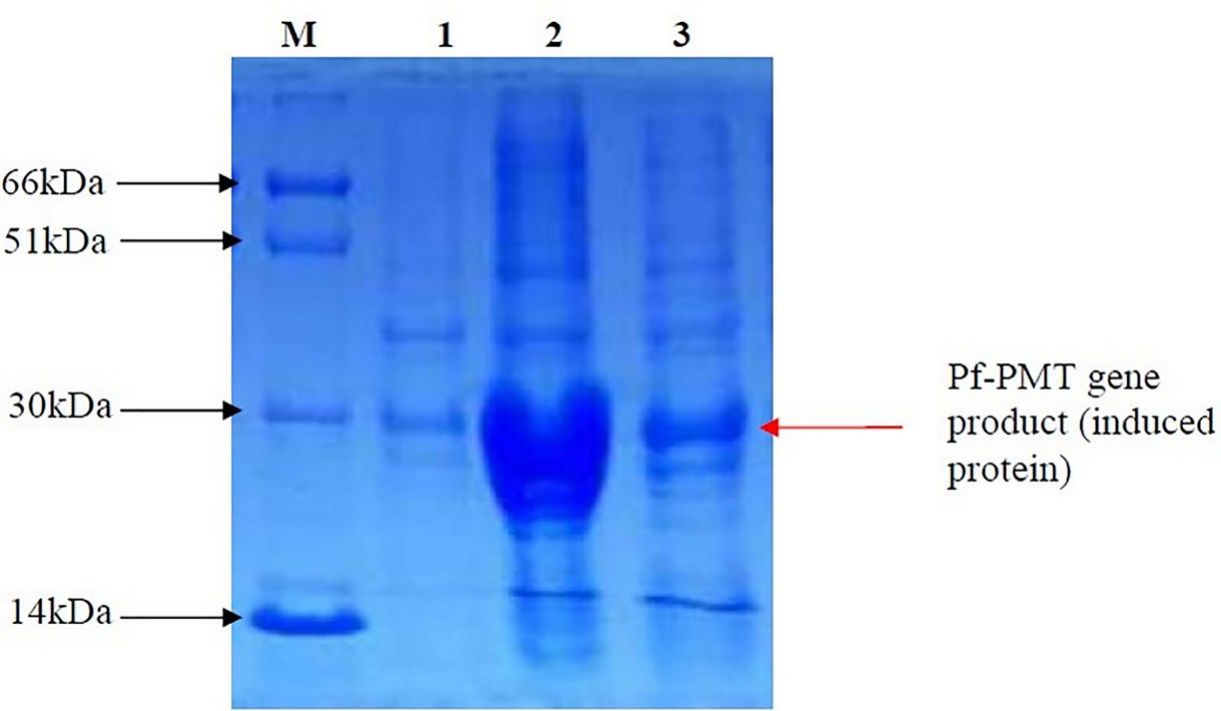
Protein Expression Checking *(Pf-*PMT*)*, Loaded on12%SDS-PAGE. (1)–Before induction sample (whole cell extract). (2)– 16 hours after induction sample (whole cell extract). (3)– 4 hours after induction sample (whole cell extract), M–Protein Marker.

*Pf*PMT was cloned using pET-24a expression vector and expressed in BL21 (DE3). Expression host was purified using gel extraction protocol and presence of purified fractions was checked on 12% SDS-PAGE. Purified protein was confirmed by MALDI TOF MS/MS analysis and recombinant *Pf*PMT protein band was observed at the expected 30 KDa (Fig 9).

Purified *Pf*PMT protein assayed using kit was found to be functional which was further used for assaying the primary hits to identify the inhibitors. This assay depends upon the transmethylation of phosphoethanolamine (pEth) into phosphocholine (pCholine) by *Pf*PMT transferring three methyls of SAM and formation of S-Adenosylhomocysteine (AdoHcy: tridemethylated SAM). Rapid degradation of AdoHcy by AdoHcy Nucleosidase gets into S-Ribosylhomocysteine and adenine where adenine deaminase acts on adenine produces hypoxanthine which is rapidly gets converted into urate and hydrogen peroxide (H_2_O_2_) by xanthine oxidase. Rate of production of H_2_O_2_ was measured by increase in absorbance at 510 nm with the help of colorimetric reagent 3,5-dichloro-2-hydroxybenzensulfonic acid (DHBS).

**Fig 10:**
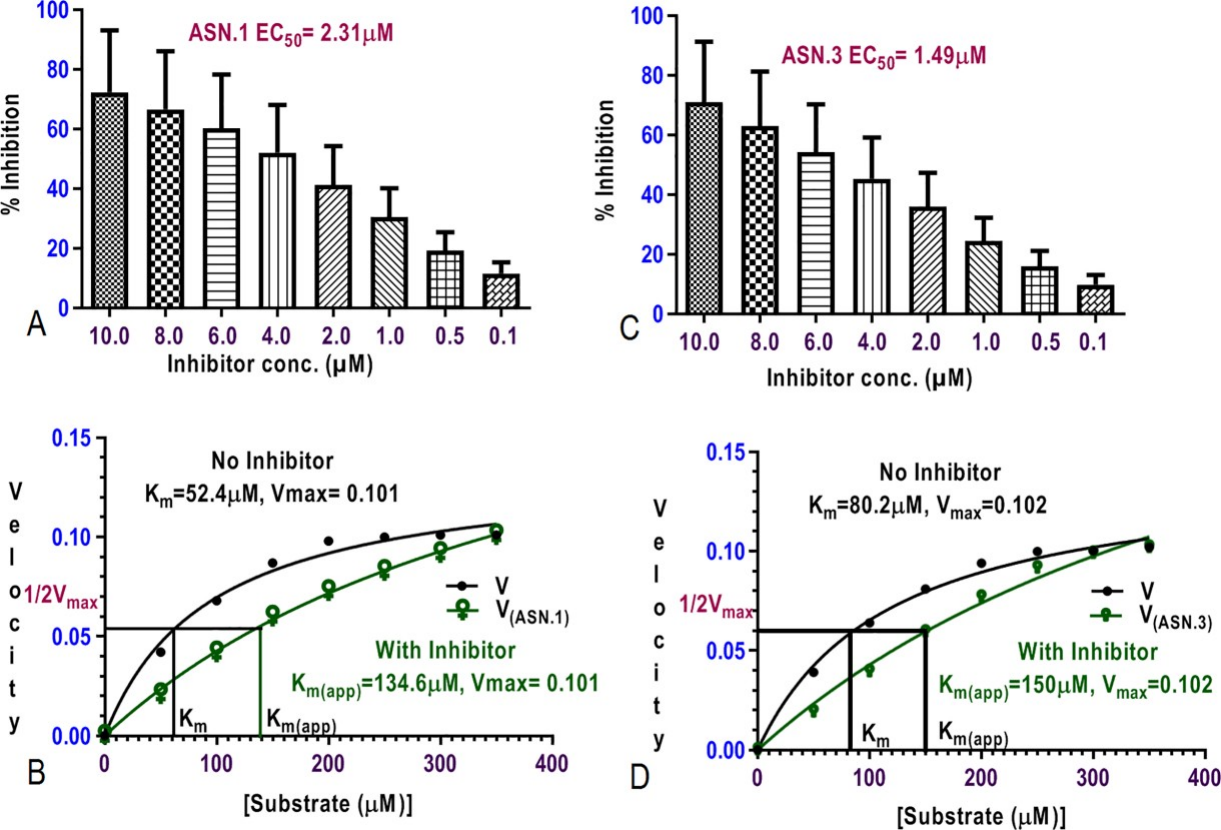
SAM dependent inhibitor assay and kinetics of *Pf*PMT at 510nm (A) EC_50_ of ASN.1 inhibitor against *Pf*PMT with P* (0.05) (C) inhibitor kinetics of the ASN.1 with 134.6 μM and (B) EC_50_ of ASN.3 inhibitor with P* (D) inhibitor kinetics of the ASN.3 with 150 μM.

*Pf*PMT assay was run over 30 minutes and increase in absorbance at 510 nm was recorded and change in absorbance was also recorded in absence of *Pf*PMT. *Pf*PMT inhibition assay to identify inhibitors was carried out at varying concentration of hits and calculated the EC_50_ of the hits using Prism GraphPad 5.0. ASN.1and ASN.3 was inhibited at very low μM conc. 1.49μM (Fig 10A) and 2.31μM (Fig 10B) with p value < 0.05 implied that these may be specific inhibitors of *Pf*PMT. Kinetics of *Pf*PMT reaction was studied by increasing the conc. of substrate pEth and Vmax and Km were calculated.

Primary hits were assayed with established *Pf*PMT assay and showed significant inhibition of *P*fPMT at 510 nm at very low micromolar concentration. For the kinetics of these primary hits, the hits concentration was kept fixed at varying concentrationof substrate. The Michaelis Menten kinetic plot was plotted for experimental values to determine the mode of *Pf*PMT inhibition by inhibitors. Vmax and Km values of *Pf*PMT from experimental values were calculated in presence and absence of inhibitors and correlated to calculate the Ki values of inhibitors (Fig 10). In presence of inhibitor, ASN.1 [K_m(app)_] was found 134.2 μM much higher than (K_m_) in absence of inhibitor (52.4 μM) at equal V_max_ (0.101) whereas K_i_ (Inhibition constant) was found to be 1.2 μM. K_m(app)_ and K_m_ of inhibitor which is less than the EC_50_ of *Pf*PMT (Fig 10C). [K_m(app)_] and(K_m_) for ASN.3 was also found 150μM and 80.2μM respectively (Fig 10D) at equal V_max_ (0.102) with K_i_ value 2.2 μM which is again less than the EC_50_ of *Pf*PMT (Table 3). Inhibitors which had V_max_ equal to the substrate pEthanolamine and as these inhibitors were interacting with the crucial amino acids within the substrate binding cavity in the binding orientation similar to the substrate that signified the competitive mode of inhibition of ASN.1.

#### *In vivo* efficacy

The primary hits with good inhibitory activity and safety index were also studied for efficacy in mice in terms of % growth inhibition of parasitaemia. The mean % parasitaemia and % growth inhibition of parasite of *Pf*PMT inhibitors was calculated based on the fifth day of dosing at 50mg/Kg, 10mg/Kg oral dosage and 5mg/Kg, 1mg/Kg IV dosage. The inhibitors which reduced the parasite growth ≥30% were considered as active and data of two tested inhibitors found active against *P*. *berghei* presented (Table 4). The groups of oral dose (50mg/Kg) of inhibitors reduced parasite growth more than 30% where ASN.1 reduced more than 50% of the parasite growth and also showed significant growth inhibition (32.5%) at 10mg/Kg. Intravenous (IV) dosage of this inhibitor greatly affected the parasite growth with 73.1% and 59.8% inhibition at 5mg/Kg and 1mg/Kg IV dosage respectively. Inhibitor ASN.3 also showed the significant activity at 50mg/Kg with 30.36% growth inhibition and both IV dosages with highest activity 49.4% growth inhibition at 5mg/Kg IV dosage. ASN.1 showed good potency both at oral as well as IV dosages implied the better druglikeness but ASN.3 could only reduce the growth of parasite better at IV dosages that may be because of pharmacokinetic problem of the ASN.3 which can be optimized and improved. Hence, the schizonticidal activities of both the *Pf*PMT inhibitors confirmed the good *in*-*vivo* antimalarial potency at lower micromolar concentration.

**Table 4:** Efficacy of *Pf*PMT inhibitors in *P*. *berghei*infected mice in terms of % growth inhibition.

Day	Day 5^th^ (Oral)				Day 5^th^ (IV)			
Dosage	50mg/Kg (Oral)		10mg/Kg (Oral)		5mg/Kg (IV)		1mg/Kg (IV)	
	% Parasitemia	% Growth inhibition	% Parasitemia	% Growth inhibition	% Parasitemia	% Growth inhibition	% Parasitemia	% Growth inhibition
**ASN.1**	14.2±1.73	55.06	21.3±1.9	32.5	8.5±0.31	73.1	12.7±1.32	59.8
**ASN.3**	21.5±0.84	30.36	27.6±1.7	12.6	15.2±1.0	49.4	18.1± 0.22	42.05

Negative control (**% parasitaemia) =** 0.012±0.0

Positive control (**% parasitaemia) =** 31.6±0.48

### Conclusion

The continuous usage of first line of antimalarial drugs often leads to resistance problem calls for the need for search and novel drug development. New drug development is however difficult but identification of a potential drug target is essential for structure based drug development. The *Pf*PMT needed for PC synthesis through SDPM pathway during both intraerythrocytic cycle and gametocyte development fulfills the urge of plausible drug target and use it for structure based drug development of new antimalarial.

The amplified Ind_*Pf*PMT was found to be fully conserved in comparison with *Pf*PMT 3D7 strain. The significant domain identification and sequence conservation was also found with human infecting Plasmodium orthologues more importantly among AdoMet binding and phosphobase domains. The phylogenetic analysis revealed the close evolutionary relatedness with functional as well as structural conservation among plasmodium. The human PEMT isoforms were found to be evolved independently and distantly. Hence, the enzyme *Pf*PMT has been identified as an important drug targetwhich may be used to develop common antimalarial against human infecting Plasmodium species worldwide.

The druglike compounds procured based on affinity sorted based on interaction affinity with target protein and crucial amino acids for biological function of protein. The schizonticidal testing provided three primary hits ASN.1, ASN.2 and ASN.3 with IC_50_ 1.77μM, 3.93μM and 3.21μM respectively, and significant gametocidal activity with 3.1μM, 6.2μM and 3.8μM respectively below the five μM concentrations. The primary hits ASN.1 and ASN.3 with heterocyclic furan, pyridine, and pyrimidine chemo types were found nontoxic to HEK-293 cells. The *Pf*PMT was inhibited at very low μM concentration and the protein kinetics demonstrated the competitive mode of inhibition of ASN.1 and ASN.3. The IV dose of both ASN.1 and ASN.3 showed good growth inhibition of *P*. *Berghei* in mice. The 50mg/kg oral dosing of ASN.1 and ASN.3 implied the good *in vivo* efficacy for *P*. *beghei*. Hence, *Pf*PMT inhibitors ASN.1 and ASN.3 have been found to have good druglike properties and interaction with crucial amino acids. In conclusion, these inhibitors have good probability to be a good antimalarial and may be optimized to improve the bioactivity and may also be used as template for structure based drug designing.

## Supporting information

S1 Protocol Checklist_Arrive_PLOS. The protocol designed and implemented to carry out the *in vivo* experiments in mice.
(DOC)

## References

[1] World report on malaria. World Health Organization, 2018.

[2] Pascal R, Kamini M, Amy B. Management of Antimalarial Drug Resistance for the Roll Back Malaria (RBM). Antimalarial drug resistance strategy 17^th^ meeting; 2009.

[3] Lykidis A, Jackowski S. Regulation of mammalian cell membrane biosynthesis. Prog. Nucleic Acid Res. Mol. Biol. 2001; 65:361–393. 11008493 10.1016/s0079-6603(00)65010-9

[4] Pessi G, Kociubinski G, Mamoun CB. A pathway for phosphatidylcholine biosynthesis in Plasmodium falciparum involving phosphoethanolamine methylation. Proc Natl Acad Sci U S A. 2004; 101(16):6206–6211. 10.1073/pnas.0307742101 15073329 PMC395947

[5] Comerci DJ, Altabe S, de Mendoza D, Ugalde RA. Brucella abortus synthesizes phosphatidylcholine from choline provided by the host. J. Bacteriol. 2006; 188:1929–1934. 10.1128/JB.188.5.1929-1934.2006 16484204 PMC1426538

[6] Conover GM, Martinez-Morales F, Heidtman MI, Luo ZQ, Tang M, Chen C, et al Phosphatidylcholine synthesis is required for optimal function of Legionella pneumophila virulence determinants. Cell. Microbiol. 2008; 10:514–528. 10.1111/j.1462-5822.2007.01066.x 17979985 PMC2700552

[7] Meriyem AMW, Stephanie H, Sonja K, Jan G, Franz N. Phosphatidylcholine biosynthesis and its significance in bacteria interacting with eukaryotic cells. Eur. J. Cell. Biol. 2010; 89:888–894. 10.1016/j.ejcb.2010.06.013 20656373

[8] Gabriella P, Jae YC, Jennifer MR, Dennis RV, Choukri BM. In Vivo Evidence for the Specificity of Plasmodium falciparum Phosphoethanolamine Methyltransferase and Its Coupling to the Kennedy Pathway. J. Biol. Chem. 2005; 280(13): 12461–12466. 10.1074/jbc.M414626200 15664981

[9] Reynolds JM, Takebe S, Choi JY, Bissati KEL, Witola WH, Bobenchik AM, et al Biochemical and Genetic Analysis of the Phosphoethanolamine Methyltransferase of the Human Malaria Parasite Plasmodium falciparum. J. Biol. Chem. 2008; 283(12):7894–7900. 10.1074/jbc.M709869200 18178564

[10] Bobenchik AM, Witola WH, Augagneur Y, Nic Lochlainn L, Garg A, Pachikara N, et al Plasmodium falciparum phosphoethanolamine methyltransferase is essential for malaria transmission. Proc Natl Acad Sci. 2013; 110 (45):18262–18267. 10.1073/pnas.1313965110 24145416 PMC3831454

[11] Nuccio ML, Ziemak MJ, Henry SA, Weretilnyk EA, Hanson AD. cDNA cloning of phosphoethanolamine N-methyltransferase from spinach by complementation in Schizosaccharomyces pombe and characterization of the recombinant enzyme. J. Biol. Chem. 2000; 275:14095–14101. 10.1074/jbc.275.19.14095 10799484

[12] Bolognese CP, McGraw P. The isolation and characterization in yeast of a gene for Arabidopsis S-adenosylmethionine phosphor-ethanolamine N-methyltransferase. Plant Physiol. 2000; 124:1800–1813. 10.1104/pp.124.4.1800 11115895 PMC59876

[13] Charron JB, Breton G, Danyluk J, Muzac I, Ibrahim RK, Sarhan F. Molecular and biochemical characterization of a cold-regulated phosphoethanolamine N-methyltransferase from wheat. Plant Physiol. 2002; 129:363–373. 10.1104/pp.001776 12011366 PMC155899

[14] Krause RGE, Goldring JPD. Phosphoethanolamine-N-methyltransferase is a potential biomarker for the diagnosis of P. knowlesi and P. falciparum malaria. PLoS One. 2018; 5:13(3). 10.1371/journal.pone.0193833 PMC5837800 29505599

[15] Witola WH, El Bissati K, Pessi G, Xie C, Roepe PD, Mamoun CB. Disruption of the Plasmodium falciparum Gene Results in a Complete Loss of Phosphatidylcholine Biosynthesis via the Serine-Decarboxylase-Phosphoethanolamine-Methyltransferase Pathway and Severe Growth and Survival Defects. J. Biol. Chem. 2008; 283(41):27636–27643. 10.1074/jbc.M804360200 18694927 PMC2562060

[16] Bobenchik AM, Augagneur Y, Hao B, Hoch JC, Ben Mamoun C. Phosphoethanolamine methyltransferases in Phosphocholine biosynthesis: functions and potential for antiparasite therapy. FEMS Microbiol. Rev. 2011; 35(4):609–619. 10.1111/j.1574-6976.2011.00267.x 21303393 PMC4107886

[17] Garg A, Lukk T, Kumar V, Choi JY, Augagneur Y, Voelker DR, et al Structure, Function and Inhibition of the Phosphoethanolamine Methyltransferases of the Human Malaria Parasites Plasmodium vivax and Plasmodium knowlesi. Sci. Rep. 2014; 5:9064. 10.1038/srep09064 PMC4357015 25761669

[18] Sanger F, Nicklen S, Coulson AR. DNA sequencing with chain-terminating inhibitors, 1977. Biotechnology. 1992; 24:104–8. 1422003

[19] Tom H. BioEdit: An important software for molecular biology. GERF Bulletin of Biosciences. 2011; 2(1):60–61.

[20] Corpet F. Multiple sequence alignment with hierarchical clustering. Nucl. Acids Res. 1988; 16 (22):10881–10890. 10.1093/nar/16.22.10881 2849754 PMC338945

[21] Tamura K, Stecher G, Peterson D, Filipski A, Kumar S. MEGA6: Molecular Evolutionary Genetics Analysis Version 6.0. Mol. Biol. Evol. 2013; 30(12):2725–2729. 10.1093/molbev/mst197 24132122 PMC3840312

[22] Liu J, Rost B. Domains, motifs and clusters in the proteins universe. CurrOpin Chem Biol. 2003; 7(1):5–11. 10.1016/s1367-5931(02)00003-0 12547420

[23] Puntervoll P, Linding R, Gemünd C, Chabanis-Davidson S, Mattingsdal M, Cameron S, et al ELM server: A new resource for investigating short functional sites in modular eukaryotic proteins. Nucleic acids res. 2003; 31:3625–3630. 10.1093/nar/gkg545 12824381 PMC168952

[24] Mitchell A, Chang HY, Daugherty L, Fraser M, Hunter S, Lopez R, et al The Interpro protein families’ database: the classification resources after 15 years. Nucleic Acids Res. 2015; 28:D213–D21. 10.1093/nar/gku1243 PMC4383996 25428371

[25] Lee SG, Kim Y, Alpert TD, Nagata A, Jez JM. Structure and Reaction Mechanism of Phosphoethanolamine Methyltransferase from the Malaria Parasite Plasmodium falciparum: AN ANTIPARASITIC DRUG TARGET. J. Biol. Chem. 2012; 287:1426–1434. 10.1074/jbc.M111.315267 22117061 PMC3256908

[26] Chatterjee A, Cutler SJ, Doerksen RJ, Khan IA, Williamson JS. Discovery of thienoquinolone derivatives as selective and ATP non-competitive CDK5/p25 inhibitors by structure-based virtual screening. Bioorg Med Chem. 2014; 22(22): 6409–6421. 10.1016/j.bmc.2014.09.043 25438765 PMC4254530

[27] Friesner RA, Murphy RB, Repasky MP, Frye LL, Greenwood JR, Halgren TA, et al Extra precision glide: docking and scoring incorporating a model of hydrophobic enclosure for protein-ligand complexes. J Med Chem. 2006; 49:6177–96. 10.1021/jm051256o 17034125

[28] Egan WJ, Merz KM Jr, Baldwin JJ. Prediction of drug absorption using multivariate statistics. J Med Chem. 2000; 43:3867-7l7. 10.1021/jm000292e 11052792

[29] Cheng A, Merz KM Jr. Prediction of aqueous solubility of a diverse set of compounds using quantitative structure-property relationships. J Med Chem. 2003; 46:3572–80. 10.1021/jm020266b 12904062

[30] Lipinski CA. Lead- and drug-like compounds: the rule-of-five revolution. Drug Discov Today Technol. 2004; 1(4):337–41. 10.1016/j.ddtec.2004.11.007 24981612

[31] Gombar VK, Enslein K. Assessment of n-octanol/water partition coefficient: When is the assessment reliable? J Chem Inf Comput Sci. 1996; 36:1127–34. 8941993 10.1021/ci960028n

[32] Mosmann T. Rapid colorimetric assay for cellular growth and survival: application to proliferation and cytotoxicity assays. J Immunol Methods. 1983; 16, 65(1–2):55–63. 10.1016/0022-1759(83)90303-4 6606682

[33] Rieckmann KH, Campbell GH, Sax LJ, Mrema JE. Drug sensitivity of plasmodium falciparum. An in-vitro microtechnique. Lancet. 1978; 7, 1(8054):22–3. 10.1016/s0140-6736(78)90365-3 74500

[34] Singh J, Kumar M, Mansuri R, Sahoo GC, Deep A. Inhibitor designing, virtual screening, and docking studies for methyltransferase: Apotential target against dengue virus. J Pharm Bioallied Sci. 2016; 8(3):188–94. 10.4103/0975-7406.171682 27413346 PMC4929957

[35] Wadi I, Pillai CR, Anvikar AR, Sinha A, Nath M, Valecha N. Methylene blue induced morphological deformations in Plasmodium falciparum gametocytes: implications for transmission-blocking. Malar J. 2018; 17:11 10.1186/s12936-017-2153-9 29310655 PMC5759873

[36] Singh J, Mansuri R, Vijay S, Sahoo GC, Sharma A, Kumar M.Docking predictions basedPlasmodium falciparum phosphoethanolamine methyltransferase inhibitor identification and in‑vitro antimalarial activity analysis. BMC chem.2019; 13:43 1–13. 10.1186/s13065-019-0551-5 PMC6661969 31384791

[37] Selvaraj V, Bodapati S, Murray E, Rice KM, Winston N, Shokuhfar T, et al Cytotoxicity and genotoxicity caused by yttrium oxide nanoparticles in HEK293 cells. Int J Nanomedicine. 2014; 12, 9:13, 79–91. 24648735 10.2147/IJN.S52625 PMC3958544

[38] Bobenchik AM, Choi JY, Mishra A, Rujan IN, Hao B, Voelker DR, et al Identification of inhibitors of Plasmodium falciparum phosphoethanolamine methyltransferase using an enzyme-coupled transmethylation assay. BMC Biochem. 2010; 11:4 10.1186/1471-2091-11-4 20085640 PMC2824672

[39] Melariri P, Campbell W, Etusim P, Smith P. In Vitro and in Vivo Antimalarial Activity of Linolenic and Linoleic Acids and their Methyl Esters. Adv Stud Biol. 2012; 4(7):333–349.

[40] Committee for the Purpose of Control and Supervision on Experiments on Animals. CPCSEA Guidelines for Laboratory Animal Facility. Indian J. Pharmacol. 2003; 35: 257–274.

