# Supplementary material for: Computational and experimental elucidation of Plasmodium falciparum phosphoethanolamine methyltransferase inhibitors: Pivotal drug target
Source: PLoS One. 2019 Aug 22;14(8):e0221032. doi: 10.1371/journal.pone.0221032 (PMC6705855; doi:10.1371/journal.pone.0221032)
Supplement: S1 Protocol — The protocol designed and implemented to carry out the in vivo experiments in mice. (DOC) [file pone.0221032.s001.doc]

**The ARRIVE Guidelines Checklist**

**Animal Research: Reporting In Vivo Experiments**

| **SECTION/ TOPIC** | **ITEM**  **No.** | **RECOMMENDATION** | **DESCRIPTION** |
| --- | --- | --- | --- |
| **TITLE** | 1 | Provide as accurate and concise a description of the content of the article as possible. | Computational and experimental elucidation of *Plasmodium falciparum* phosphoethanolamine methyltransferase inhibitors: pivotal drug target |
| **ABSTRACT** | 2 | Provide an accurate summary of the background, research objectives including details of the species or strain of animal used, key methods, principal findings, and conclusions of the study. | - The present study, was designed to test schizonticidal and Nontoxic primary hits for antimalarial efficacy orally and intravenously. - The *Plasmodium. berghei* albino mice model was used for antimalarial testing. - The promising reduction in parasitaemia was found in both route of administration for the primary hits. - The primary hits containing Pyridinyl-Pyrimidine and Phenyl-Furan scaffolds may the possible antimalarial candidate and may further be optimized and used as lead for template based drug development. |
| **INTRODUCTION** | | | |
| **Background** | 3 | 1. Include sufficient scientific background (including relevant references to previous work) to understand the motivation and context for the study, and explain the experimental approach and rationale. 2. Explain how and why the animal species and model being used can address the scientific objectives and, where appropriate, the study’s relevance to human biology. | - In the present study, we reported the characterization and phylogenetic comparison of *Pf*PMT gene and comparative analysis showed significant identity among SAM-dependent methyltransferase domains and motifs of Plasmodium orthologues. - To delineate this protein as a drug target, we undertook docking studies to identify the compounds inhibitors of *Pf*PMT that either interacting with crucial amino acids or occupying the catalytic dyad important for triple methylation through *in vitro* and *in vivo* efficacy studies in albino mice as well. - *In vitro* analysis revealed two compounds ASN.1 & ASN.3 found to be nontoxic and competitive inhibitors of *Pf*PMT with IC_50_ 1.49µM and 2.31µM respectively with significant gametocidal activity. - The *in vivo* efficacy study in albino mice showed the promising reduction in parasitaemia both in orally (50 & 10 mg/kg) and intravenous (IV) (5& 1 mg/kg) but the better growth inhibition was found in IV groups. |
| **Objectives** | 4 | Clearly describe the primary and any secondary objectives of the study, or specific hypotheses being tested. | - The present study aims to project *Pf*PMT inhibitors (primary hits) as possible antimalarials. - Two primary hits containing Pyridinyl-Pyrimidine and Phenyl-Furan scaffolds showed good *in vitro* schizonticidal and gametocidal activity were also tested in *Plasmodium. berghei* albino mice model for schizonticidal activity in mice to correlate both the *in vitro* and *in vivo* activity.. - *Pf*PMT inhibitors found active in mice provide insights into the mechanism of blocking transmission of the malaria infection and may set as an imminent prospect to overcome the problem of drug resistance. |
| **METHODS** | | | |
| **Ethical statement** | 5 | Indicate the nature of the ethical review permissions, relevant licenses (e.g. Animal [Scientific Procedures] Act 1986), and national or institutional guidelines for the care and use of animals, that cover the research. | Animal experiments were conducted [at department of Animal Experiments, National JALMA Institute for Leprosy and Other Mycobacterial diseases](https://www.jalma-icmr.org.in/) under the grant of ethical approval from the Animal Research Ethics Committee JALMA, Agra, India. (**Approval Id: JALMA/2018/10**). |
| **Study design** | 6 | For each experiment, give brief details of the study design, including:   1. The number of experimental and control groups. 2. Any steps taken to minimize the effects of subjective bias when allocating animals to treatment (e.g., randomization procedure) and when assessing results (e.g., if done, describe who was blinded and when). 3. The experimental unit (e.g. a single animal, group, or cage of animals).   A time-line diagram or flow chart can be useful to illustrate how complex study designs were carried out. | 1. One experimental group and two control groups. 2. No 3. Group (Three groups) |
| **Experimental procedures** | 7 | For each experiment and each experimental group, including controls, provide precise details of all procedures carried out. For example:   1. How (e.g., drug formulation and dose, site and route of administration, anaesthesia and analgesia used [including monitoring], surgical procedure, method of euthanasia). Provide details of any specialist equipment used, including supplier(s). 2. When (e.g., time of day). 3. Where (e.g., home cage, laboratory, water maze). 4. Why (e.g., rationale for choice of specific anaesthetic, route of administration, drug dose used). | One donor group of two mice was kept.  Experiment was divided into three groups (Test, Positive and Negative groups) with three mice in each group.   1. Dosages were calculated as per body weight and route of administration orally (gavage needle) and Intravenous (IV) and standard dose selected. Dosed at 50mg/Kg, 10mg/Kg oral dosage and 5mg/Kg, 1mg/Kg IV dosage 2. The first dose was administered on first day two hours later of the infection followed by other three doses each at 24 hours interval. 3. Laboratory 4. To estimate the oral and intravenous efficacy of test compounds. |
| **Experimental animals** | 8 | 1. Provide details of the animals used, including species, strain, sex, developmental stage (e.g., mean or median age plus age range), and weight (e.g., mean or median weight plus weight range). 2. Provide further relevant information such as the source of animals, international strain nomenclature, genetic modification status (e.g. knock-out or transgenic), genotype, health/immune status, drug- or test-naïve, previous procedures, etc. | 1. Male albino mice of twelve to sixteen months old 2. Weight ranging 22 to 25 g. 3. NA |
| **Housing and husbandry** | 9 | Provide details of:   1. Housing (e.g., type of facility, e.g., specific pathogen free (SPF); type of cage or housing; bedding material; number of cage companions; tank shape and material etc. for fish). 2. Husbandry conditions (e.g., breeding programme, light/dark cycle, temperature, quality of water etc. for fish, type of food, access to food and water, environmental enrichment).   Welfare-related assessments and interventions that were carried out before, during, or after the experiment. | - Triple distilled Millipore purified water (Millipore, USA) used to prepare solutions. - Mice were exposed to the day/night light cycle of 12 hours period. - Mice were monitored properly with plentiful dried food and water and their sanitation was carefully monitored every day. |
| **Sample size** | 10 | 1. Specify the total number or animals used in each experiment and the number of animals in each experimental group. 2. Explain how the number of animals was decided. Provide details of any sample size calculation used. 3. Indicate the number of independent replications of each experiment, if relevant. | 1. Two mice were kept in donor group, three mice in test, positive and negative groups. 2. Three mice in a single group were kept to perform test in triplicate. 3. Total of 11 mice for a single compound. |
| **Allocating animals to experimental groups** | 11 | 1. Give full details of how animals were allocated to experimental groups, including randomisation or matching if done. 2. Describe the order in which the animals in the different experimental groups were treated and assessed. | 1. In the present study, Male albino mice of twelve to sixteen months old, weight ranging 22 to 25 g used. 2. Experimental group was randomly selected. |
| **Experimental outcomes** | 12 | Clearly define the primary and secondary experimental outcomes assessed (e.g., cell death, molecular markers, behavioural changes). | - On the primary hits testing, no cell death was found. - Even no mood swing and behavior changes in study animals were found. |
| **Statistical methods** | 13 | 1. Provide details of the statistical methods used for each analysis. 2. Specify the unit of analysis for each dataset (e.g. single animal, group of animals, single neuron). 3. Describe any methods used to assess whether the data met the assumptions of the statistical approach. | The test compounds were administered after 24 hrs after first dose. At last fifth day the blood sample were studied for % parasitaemia and % growth inhibition.  Farmula’s applied  % parasitaemia **=** No. of Schizonts counted**/** _No_. of parasitized RBCs counted **X**100  % Inhibition **=** 1- No. of Schizonts in test**/** _No_. of Schizonts Positive control **X**100 |
| **RESULTS** | | | |
| **Baseline data** | 14 | For each experimental group, report relevant characteristics and health status of animals (e.g., weight, microbiological status, and drug- or test-naïve) before treatment or testing (this information can often be tabulated). | Male albino mice of twelve to sixteen months old.  Weight ranging 22 to 25 g. |
| **Numbers analysed** | 15 | 1. Report the number of animals in each group included in each analysis. Report absolute numbers (e.g., 10/20, not 50% **^*^**). 2. If any animals or data were not included in the analysis, explain why. | Three mice in a single group were kept to perform test in triplicate |
| **Outcomes and estimation** | 16 | Report the results for each analysis carried out, with a measure of precision (e.g., standard error or confidence interval). | - The primary hits were studied for efficacy in mice in terms of % growth inhibition of parasitaemia. - The mean % parasitaemia and % growth inhibition of parasite of *Pf*PMT inhibitors was calculated after the fifth day - The oral dose (50mg/Kg) of both test compounds reduced parasite growth more than 30%. - ASN.1 reduced more than 50% of the parasite growth at 50mg/Kg and 32.5% at 10mg/Kg. Intravenous (IV) dosage of this inhibitor greatly affected the parasite growth with 73.1% and 59.8% inhibition at 5mg/Kg and 1mg/Kg IV dosage respectively. - Inhibitor ASN.3 also showed the significant activity with 30.36% growth inhibition at 50mg/Kg. The IV dosage 5mg/Kg showed activity 49.4% growth inhibition. |
| **Adverse events** | 17 | 1. Give details of all important adverse events in each experimental group. 2. Describe any modifications to the experimental protocols made to reduce adverse events. | NA |
| **DISCUSSION** | | | |
| **Interpretation/scientific**  **implications** | 18 | 1. Interpret the results, taking into account the study objectives and hypotheses, current theory, and other relevant studies in the literature. 2. Comment on the study limitations including any potential sources of bias, any limitations of the animal model and the imprecision associated with the results^*^. 3. Describe any implications of your experimental methods or findings for the replacement, refinement, or reduction (the 3Rs) of the use of animals in research. | The IV dose of both ASN.1 and ASN.3 showed good growth inhibition of *P. Berghei* in mice. The 50mg/kg oral dosing of ASN.1 and ASN.3 implied the good *in vivo* efficacy for *P. beghei*. ASN.1 showed good potency both at oral as well as IV dosages implied the better druglikeness but ASN.3 could only reduce the growth of parasite better at IV dosages that may be because of pharmacokinetic problem of the ASN.3 which can be optimized and improved. Hence, the schizonticidal activities of both the *Pf*PMT inhibitors confirmed the good *in*-*vivo* antimalarial potency at lower micromolar concentration. Hence, *Pf*PMT inhibitors ASN.1 and ASN.3 found to have good druglike properties and interaction with crucial amino acids. In conclusion, these inhibitors have good probability to be a good antimalarial and may be optimized to improve the bioactivity and may also be used as template for structure based drug designing |
| **Generalisability/ translation** | 19 | Comment on whether, and how, the findings of this study are likely to translate to other species or systems, including any relevance to human biology. | The *in vivo* efficacy for *P. beghei* delivered the two leads with heterocyclic furan, pyridine, and pyrimidine chemo types. These leads have good probability to be a good antimalarial and may be optimized to improve the bioactivity and may also be used as template for structure based drug designing. |
| **Funding** | 20 | List all funding sources (including grant number) and the role of the funder(s) in the study | Study was funded by ICMR through Intramural grant (Id: **NIMR/PB/2015/310)** from NIMR, Dwarka sector-8(ICMR). |
